# Increased risk of bleeding during and after HoLEP in patients with prostate cancer: A multicentre comparative cohort study

**DOI:** 10.1002/bco2.70060

**Published:** 2025-09-16

**Authors:** Angelo Porreca, Filippo Marino, Davide De Marchi, Marco Giampaoli, Francesca Simonetti, Antonio Amodeo, Paolo Corsi, Francesco Claps, Alessandro Crestani, Gian Maria Busetto, Daniele D'Agostino, Daniele Romagnoli, Luca Di Gianfrancesco

**Affiliations:** ^1^ Department of Urology Humanitas Gavazzeni Bergamo Italy; ^2^ Veneto Institute of Oncology IOV – IRCCS Padua Italy; ^3^ Department of Urology Azienda Sanitaria Universitaria Friuli Centrale Udine Italy; ^4^ Department of Urology Ospedali Riuniti di Foggia, Università di Foggia Foggia Italy; ^5^ Department of Urology Villa Salus Mestre Italy; ^6^ Department of Urology Policlinico Abano Terme Abano Terme Italy

**Keywords:** antithrombotic therapy, bleeding complications, HoLEP, prostate cancer, retrospective cohort study, transfusion

## Abstract

**Objective:**

To assess the frequency and severity of bleeding complications during and after Holmium Laser Enucleation of the Prostate (HoLEP) in patients with prostate cancer, and compare outcomes to a control group of patients without prostate cancer but with similar baseline characteristics.

**Methods:**

This multicentre retrospective study included 175 consecutive patients undergoing HoLEP across 3 referral centres with a diagnosis of prostate cancer—128 with known cancer prior to surgery and 47 with incidental findings on postoperative histology. These patients were compared to 500 consecutive control patients without prostate cancer but matched for prostate volume, age, presence of indwelling catheter, comorbidities and anticoagulant/antiplatelet therapy status. Bleeding‐related events analysed included intraoperative estimated blood loss, need for transfusion, clot retention, postoperative irrigation, reoperation for haemorrhage and hospital readmission within 30 days.

**Results:**

The PCa group experienced significantly higher rates of intraoperative bleeding requiring intensified coagulation (18.3% vs 8.6%, *p* < 0.01), transfusion (6.3% vs 2.0%, *p* = 0.02) and clot retention (4.0% vs 1.4%, *p* = 0.04) compared to controls. Among patients with known PCa, 25.0% experienced bleeding‐related complications, while the rate was 14.9% among those with incidental PCa. Patients with a known diagnosis showed higher bleeding risk than incidental cases. In multivariate analysis, both prostate cancer and anticoagulant therapy were independently associated with increased risk of bleeding complications. Antithrombotic/antiplatelet therapy significantly raised the likelihood of bleeding events (adjusted OR 2.8, 95% CI 1.6–4.7; p < 0.001), as did the presence of prostate cancer (adjusted OR 2.1, 95% CI 1.3–3.6; p = 0.004). Patients with both risk factors experienced the highest rate of bleeding (29.4%), compared to 8.1% in those without either factor (p < 0.001), indicating a synergistic effect. No significant differences were found in catheter removal time or hospital stay.

**Conclusions:**

Prostate cancer—particularly when known preoperatively—is associated with a significantly increased risk of bleeding during and after HoLEP, even when controlling for baseline characteristics. Surgeons should anticipate increased vascularity and plan perioperative management accordingly to mitigate haemorrhagic complications.

## 1 INTRODUCTION

Holmium Laser Enucleation of the Prostate (HoLEP) is widely recognized as a size‐independent gold‐standard for the surgical management of benign prostatic hyperplasia (BPH), offering excellent long‐term outcomes and superior safety profiles compared to traditional methods such as transurethral resection of the prostate (TURP).^1^ The holmium: YAG laser facilitates effective haemostasis by sealing small and medium vessels up to 2–3 mm deep, which minimizes intraoperative and postoperative bleeding risks.^2^

Clinical trials and meta‐analyses confirm that HoLEP results in lower haemoglobin drop, reduced transfusion rates and shorter catheterization and hospitalization durations compared to TURP and open prostatectomy.^3^ A pivotal review by Wroclawski et al. demonstrated consistent haemostatic advantages of HoLEP regardless of prostate size.^4^ These benefits extend to patients on antiplatelet or anticoagulant therapy, with evidence supporting HoLEP's favourable bleeding profile even in these higher‐risk populations.^5^

Despite the widespread safety of HoLEP, bleeding complications—though uncommon—still present clinical challenges. In a retrospective review of 130 consecutive HoLEP procedures, 6.7% of patients required perioperative blood transfusion and 1 case was attributed to previously undiagnosed prostate cancer.^6^ Additionally, a large multicentre series involving 963 patients reported a perioperative transfusion rate of 5%. In this study, although patients on antithrombotic therapy showed longer catheterization times and hospital stays, there was no significant difference in haemoglobin drop or overall blood loss compared to patients not on such therapy.^7^

**FIGURE 1:**
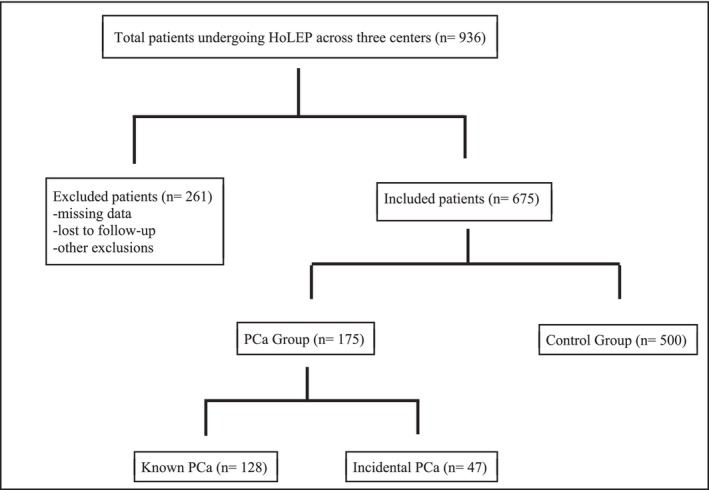
Patients selection flow diagram.

The interaction between HoLEP and prostate cancer (PCa) is still under‐explored. Prostate carcinoma is known to exhibit increased microvascular density—approximately double that of benign tissue in immunohistochemical analyses—indicating elevated blood flow in malignant areas.^8^ This increased vascularity, along with possible distortion of anatomical dissection planes, may enhance bleeding risk during enucleation. Although a notable advantage of HoLEP is its ability to provide tissue for histopathology—thereby identifying incidental PCa and guiding management—evidence quantifying perioperative bleeding in this specific subgroup remains sparse. Consequently, the true impact of cancer‐related vascular changes on bleeding risk during and after HoLEP requires further investigation (Figure 1).

To address this evidence gap, we conducted a multicentre, comparative study involving 175 patients with prostate cancer (both known and incidental) undergoing HoLEP, and compared their bleeding outcomes to 500 matched controls without PCa. By analysing intraoperative blood loss, transfusion rates, clot retention and reoperation incidence, this study aims to quantify the impact of prostate cancer on bleeding risk and inform perioperative strategies.

## 2 MATERIALS AND METHODS

### 2.1 Study design and population

This was a retrospective, multicentre cohort study conducted across three tertiary referral centres between January 2018 and December 2023. The study included 175 consecutive male patients with histologically confirmed prostate cancer (PCa) who underwent Holmium Laser Enucleation of the Prostate (HoLEP) for lower urinary tract symptoms (LUTS) secondary to bladder outlet obstruction. Of these, 128 patients (73.1%) had a known diagnosis of PCa prior to surgery, while 47 patients (26.9%) were diagnosed with incidental PCa based on final histopathological analysis of enucleated tissue.

A control group of 500 consecutive male patients without a diagnosis of PCa, matched for baseline characteristics including age, prostate volume (measured via transrectal ultrasound or MRI), presence of indwelling urinary catheter, Charlson Comorbidity Index (CCI) and antithrombotic (anticoagulant or antiplatelet) therapy status, was selected from the same time period and institutions.

A total of 35 patients were excluded from the analysis: 20 due to incomplete clinical or histopathological data, 10 for previous prostate surgery and 5 for concurrent malignancies or other contraindications to HoLEP. Eligibility criteria included adult men undergoing HoLEP for lower urinary tract symptoms, with exclusions applied as above. Patient selection was consecutive to minimize selection bias and enhance the generalizability of findings to similar clinical settings.

Inclusion Criteria

- Adult male patients (age ≥ 18 years) undergoing Holmium Laser Enucleation of the Prostate (HoLEP) for lower urinary tract symptoms (LUTS) related to benign prostatic enlargement.
- Patients with histologically confirmed prostate cancer (PCa), either diagnosed preoperatively or incidentally found on final HoLEP specimen pathology.
- Control patients without a diagnosis of PCa, matched for baseline characteristics such as age, prostate volume, comorbidities, catheter dependency and anticoagulant/antiplatelet therapy.
- Availability of complete clinical, operative and histopathological data necessary for analysis.

Exclusion Criteria

- Patients with prior prostate surgery (e.g., TURP, open prostatectomy).
- Patients with incomplete clinical or pathological records.
- Patients with concurrent urological malignancies other than PCa.
- Patients who underwent HoLEP for indications other than benign prostatic obstruction or suspected PCa.
- Patients with contraindications to HoLEP, including active urinary tract infection or coagulopathy not corrected prior to surgery.

### 2.2 Surgical technique

All patients underwent HoLEP performed by experienced urologists (>200 HoLEP cases each), using standardized enucleation techniques (three‐lobe, T‐L technique^9^). A two‐pedal Holmium:YAG laser system (120–60 W; VersaPulse, Lumenis Ltd., Yokneam, Israel) was employed as the energy source, utilizing a 550‐μm end‐firing laser fibre (SlimLine™ 550, Lumenis Inc.). The procedure was conducted using a 26 Fr continuous‐flow resectoscope equipped with a laser bridge and a 30° lens (Karl Storz Endoscopy, CA, USA).

Following complete enucleation and relocation of the prostatic lobes into the bladder, meticulous haemostasis of the prostatic fossa was performed. This was primarily achieved using the Holmium laser set to 60 W in long‐pulse mode. In rare cases where laser coagulation proved insufficient, monopolar or bipolar resectoscopes were employed to complete haemostasis.

The enucleated prostatic tissue was then extracted using a morcellator system (VersaCut, Lumenis or Hawk® JAWS), introduced through a nephroscope (Karl Storz Endoscopy).

### 2.3 Management of Antithrombotic and Antiplatelet Therapy

In all cases, antithrombotic and antiplatelet therapies were managed in accordance with international perioperative guidelines, including the 2022 American College of Cardiology and European Association of Urology recommendations. Where feasible, anticoagulants were temporarily discontinued preoperatively and replaced with bridging low‐molecular‐weight heparin when indicated, based on individual thromboembolic risk assessments. Antiplatelet agents were held or continued selectively in consultation with cardiology, particularly in patients with recent coronary stenting or high cardiovascular risk.

### 2.4 Data collection

Electronic medical records were reviewed for demographic and clinical variables, including age, prostate volume, CCI, use of anticoagulants or antiplatelets, duration of catheterization and operative details. Histopathological reports were used to confirm cancer status. Bleeding‐related variables were collected prospectively in surgical logs and included:

- Estimated intraoperative blood loss
- Intraoperative need for additional haemostasis (beyond standard laser coagulation) (intended as the need for the operator to perform a double haemostasis step, for example, before and after the morcellation phase, or to use the bipolar resector in order to optimize haemostasis)
- Postoperative transfusion requirement
- Clot retention events (defined as bladder irrigation failure or need for clot evacuation)
- Reoperation for haemorrhage (within 30 days)
- Readmission within 30 days due to bleeding‐related complications

### 2.5 Outcomes

The primary outcome was the incidence of bleeding complications, defined as any of the following: need for transfusion, clot retention requiring intervention or surgical re‐exploration for bleeding. Secondary outcomes included duration of postoperative bladder irrigation, catheterization time, length of hospital stay and 30‐day readmission rates.

### 2.6 Complications

All postoperative complications occurring within 30 days of the procedure were prospectively recorded and retrospectively reviewed. Complications were categorized using the Clavien‐Dindo classification system, which stratifies postoperative adverse events based on the therapeutic intervention required. Grade I complications included minor events not requiring pharmacological or surgical intervention (e.g., transient haematuria managed with increased irrigation). Grade II included complications requiring pharmacologic treatment such as antibiotics or blood transfusion. Grade IIIa and IIIb denoted complications requiring surgical, endoscopic or radiologic intervention without or with general anaesthesia, respectively—for example, clot evacuation under anaesthesia. Grades IV and V indicated life‐threatening complications requiring intensive care or death, respectively. For each patient, the most severe complication within 30 days postoperatively was recorded. Complication data were independently verified at each centre by cross‐referencing clinical notes, nursing reports and hospital discharge summaries to ensure classification accuracy and consistency.

### 2.7 Statistical analysis

Descriptive statistics were reported as mean ± standard deviation (SD) or median (interquartile range) for continuous variables, and as proportions for categorical variables. Between‐group comparisons were made using Student's t‐test or Mann–Whitney U test for continuous variables and Chi‐square or Fisher's exact test for categorical data. Multivariate logistic regression was used to identify independent predictors of bleeding complications. A *p*‐value <0.05 was considered statistically significant. Statistical analyses were conducted using SPSS version 27.0 (IBM Corp, Armonk, NY).

### 2.8 Sample size calculation and statistical power

Prior to data analysis, a sample size calculation was performed to ensure adequate power to detect clinically meaningful differences in bleeding complications between patients with prostate cancer (PCa) undergoing HoLEP and matched controls without PCa.

Based on previous literature indicating a transfusion rate of approximately 2% in non‐PCa patients and up to 6% in PCa patients undergoing HoLEP, we estimated a minimum difference in transfusion rates of 4% to be clinically relevant (Hammerer et al., 2017; Shah et al., 2019).

Using a two‐sided chi‐square test with α = 0.05 and aiming for 80% power (β = 0.20), the required sample size per group to detect this difference was calculated as follows:

- Estimated transfusion rate in controls (p1): 0.02
- Estimated transfusion rate in PCa group (p2): 0.06
- Effect size (difference): 0.04

The calculation yielded a required sample size of approximately 430 patients per group. The actual sample included 175 PCa patients and 500 controls, which provided sufficient power to detect differences in more frequent bleeding events such as intraoperative bleeding requiring intensified coagulation (observed at 18.3% vs 8.6%).

Post‐hoc power analysis confirmed that the study had >80% power to detect differences in primary outcomes including transfusion rates and clot retention, considering the observed event rates.

## 3 RESULTS

Of the 675 patients analysed, 175 had histologically confirmed prostate cancer (PCa) and 500 served as controls without PCa. Baseline characteristics, including age, prostate volume, comorbidity index, presence of an indwelling catheter and use of antithrombotic therapy, were comparable between the groups (Table 1).

**TABLE 1:** Baseline characteristics and outcomes.

Characteristic	PCa group (n = 175)	Control group (n = 500)	p‐value
Age (years), mean ± SD	69.0 ± 6.7	68.9 ± 6.7	0.92
Prostate volume (mL), mean ± SD	66.2 ± 18.3	63.9 ± 19.1	0.12
Charlson Comorbidity Index (CCI), median (IQR)	3.0 (2–4)	3.0 (3–4)	0.88
Indwelling catheter (%)	24.6%	23.0%	0.65
Anticoagulant/antiplatelet therapy (%)	18.3%	22.3%	0.21
PSA (ng/mL), mean ± SD	7.52 ± 3.43	1.41 ± 0.93	<0.001
Outcome			p‐value
Intraoperative bleeding	18.3%	8.6%	<0.01
Blood transfusion	6.3%	2.0%	0.02
Clot retention	4.0%	1.4%	0.04
Known PCa bleeding complications	25.0%	–	–
Incidental PCa complications	14.9%	–	–
Catheter removal time	No difference	No difference	>0.05
Hospital stay	No difference	No difference	>0.05

Age, Prostate Volume, PSA: two‐sample t‐test; CCI: Mann–Whitney U test; Indwelling Catheter, Anticoagulant use: Chi‐square test.

### 3.1 Bleeding outcomes

The PCa group demonstrated significantly higher rates of intraoperative bleeding requiring intensified laser coagulation compared to controls (18.3% vs. 8.6%, p < 0.01). Similarly, postoperative blood transfusion was more frequent in the PCa cohort (6.3% vs. 2.0%, p = 0.02). Clot retention events, defined as bladder irrigation failure or need for clot evacuation, occurred in 4.0% of PCa patients versus 1.4% in controls (p = 0.04).

Subgroup analysis revealed that among patients with a known diagnosis of PCa, the rate of bleeding‐related complications was 25.0%, significantly higher than the 14.9% observed in those with incidental PCa. This difference suggests that preoperatively diagnosed malignancy, which may reflect more advanced or vascularized tumour characteristics, contributes to a greater bleeding risk.

### 3.2 Predictors of bleeding

Multivariate logistic regression analysis identified both anticoagulant/antiplatelet therapy and the presence of prostate cancer as independent predictors of bleeding‐related complications. Patients on antithrombotic therapy had a significantly increased risk of intraoperative or postoperative bleeding (adjusted odds ratio [aOR] 2.8; 95% CI: 1.6–4.7; p < 0.001), regardless of PCa status. Similarly, the presence of prostate cancer—particularly in patients with a preoperative diagnosis—was associated with a higher bleeding risk (aOR 2.1; 95% CI: 1.3–3.6; p = 0.004). Notably, the combination of both risk factors (PCa + antithrombotic use) conferred an even greater risk, with an observed bleeding complication rate of 29.4% in this subgroup, compared to 8.1% in patients without either factor (p < 0.001). These findings suggest a cumulative effect and underscore the need for heightened intraoperative vigilance and individualized perioperative planning in this high‐risk population.

Prostate volume and age were not significant predictors when adjusted for comorbidities and PCa status.

### 3.3 Other perioperative outcomes

No statistically significant differences were found between groups regarding duration of postoperative bladder irrigation, time to catheter removal or length of hospital stay (all *p* > 0.05).

There were no statistically significant differences between the prostate cancer (PCa) group and the control group in terms of catheter removal time or hospital stay. The median catheterization time was 2.1 days (IQR: 1.9–2.5) for PCa patients and 2.0 days (IQR: 1.8–2.4) for controls (p = 0.12). Similarly, the mean duration of hospital stay was 2.7 ± 0.8 days for PCa patients versus 2.6 ± 0.7 days for controls (p = 0.18). Subgroup analysis revealed no significant differences between patients with known versus incidental prostate cancer regarding these parameters. These findings suggest that the presence of prostate cancer does not prolong postoperative recovery in terms of catheter dependence or hospitalization when treated with HoLEP.

The overall 30‐day readmission rate was significantly higher in the PCa group compared to controls. Specifically, 7.4% (13/175) of patients in the PCa group required readmission within 30 days of surgery, versus 3.2% (16/500) in the control group (p = 0.03). The most common reasons for readmission in the PCa cohort included delayed haematuria requiring observation or intervention (n = 6), urinary tract infection (n = 4) and clot retention (n = 3). Among patients with a known diagnosis of prostate cancer, the readmission rate was 9.4%, compared to.

## 4 DISCUSSION

This multicentre retrospective study investigated bleeding complications during and after Holmium Laser Enucleation of the Prostate (HoLEP) in patients with histologically confirmed prostate cancer (PCa), compared with matched controls without PCa. Our findings indicate that patients with PCa undergoing HoLEP have significantly higher rates of intraoperative bleeding requiring intensified coagulation, transfusions and postoperative clot retention. Moreover, those with a known diagnosis of PCa exhibited greater bleeding risk than patients with incidental PCa discovered only on final histopathology. Anticoagulant therapy emerged as an independent predictor of haemorrhagic events, confirming its known role in surgical bleeding risk (Table 2).

**TABLE 2:** Summary table outlining the key bleeding risks associated with HoLEP in prostate cancer patients.

Risk factor	Mechanism	Impact	Management strategy
**Prostate Cancer Involvement**	Increased vascularity, distorted anatomy	Higher intraoperative blood loss	Careful dissection, enhanced coagulation
**Large Prostate Volume (>100 cc)**	Longer operative time, more enucleation surface	Increased bleeding risk	Experienced surgeon, staged resection if needed
**Antithrombotic Therapy (e.g., aspirin, DOACs)**	Impaired haemostasis	Post‐op haematuria, clot retention	Adjust medication perioperatively
**Advanced Age or Comorbidities**	Reduced physiological reserve	Poor tolerance to blood loss, slower recovery	Pre‐op optimization, close monitoring
**Prior Radiation or Surgery**	Fibrosis and neovascularity	Difficult haemostasis	Anticipate prolonged procedure, laser precision
**Delayed Recognition of Bleeding**	Inadequate early monitoring	Transfusion need, reoperation	Frequent vitals, haemoglobin checks, urine colour

The association of increased bleeding in PCa patients can be explained by several pathophysiological mechanisms. Prostate cancer tissue often exhibits increased microvascular density and neoangiogenesis,^10^, ^11^ which can lead to a more fragile and highly vascularized prostate gland. This increased vascularity may complicate the enucleation process by distorting normal anatomical planes and increasing the likelihood of injuring blood vessels during tissue dissection. Similar observations have been reported in studies evaluating bleeding risks in transurethral resection of the prostate (TURP) for cancer patients.^12^ However, limited data exist specifically regarding HoLEP in the context of PCa, making our findings particularly relevant.

HoLEP has become a preferred surgical option for benign prostatic hyperplasia (BPH) due to its efficacy and favourable safety profile, including low bleeding rates and transfusion requirements compared with TURP.^13^, ^14^ A large multicentre study by Shah et al. reported an overall transfusion rate of 5% in 963 HoLEP patients, with antithrombotic therapy associated with prolonged catheter time but not significantly affecting blood loss.^15^ Our study confirms similar rates in controls but highlights an increased bleeding risk in PCa patients, suggesting that the presence of malignancy itself may be an additional bleeding risk factor.

Importantly, HoLEP offers the advantage of tissue retrieval for histopathological examination, facilitating incidental PCa diagnosis.^16^ In our cohort, 27% of PCa cases were incidental, aligning with prior reports of incidental PCa detection rates ranging from 5% to 30% after HoLEP.^17^, ^18^ These patients experienced lower bleeding complication rates compared with those with known PCa, possibly reflecting smaller tumour burden or less aggressive disease.

Anticoagulant and antiplatelet therapy have consistently been associated with increased bleeding risks in urologic surgery.^19^ Our data reaffirm this association, with anticoagulant use independently predicting haemorrhagic complications. Careful perioperative management of these medications remains crucial to balance bleeding and thromboembolic risks.

The absence of differences in catheter removal time and length of hospital stay between groups suggests that, despite increased bleeding in PCa patients, HoLEP remains a safe and feasible procedure in this population. Nonetheless, heightened intraoperative vigilance and preparedness for bleeding control are warranted when operating on PCa patients.

In comparison to Transurethral Resection of the Prostate (TURP)—the traditional gold standard—HoLEP offers superior outcomes in terms of reduced bleeding, shorter catheterization time and lower retreatment rates. A meta‐analysis by Tan et al. found that HoLEP was associated with a significantly lower transfusion rate and hospital stay compared to TURP, without compromising functional outcomes.^20^

When compared with GreenLight photoselective vaporization (PVP), HoLEP again demonstrates advantages, particularly in providing tissue for pathological analysis—essential for diagnosing incidental prostate cancer. While PVP is associated with a favourable bleeding profile, especially in anticoagulated patients, it lacks the capacity for histological diagnosis and is less effective in very large prostates.^21^

Aquablation, a newer modality utilizing waterjet ablation under real‐time imaging, has shown promising results, particularly in terms of functional outcomes and reduced sexual side effects. However, bleeding rates requiring transfusion have been reported up to 4% in prostates >80 ml, and the inability to retrieve sufficient tissue for pathology may limit its utility in patients at risk for PCa.^22^

Open simple prostatectomy, while effective for very large prostates, carries higher morbidity, longer recovery times and greater intraoperative blood loss compared to HoLEP. Kuntz et al. demonstrated similar efficacy between open prostatectomy and HoLEP, but significantly more perioperative complications were noted in the open group.^23^

Robot‐assisted simple prostatectomy (RASP) offers excellent visualization and precise enucleation, with bleeding risks comparable to HoLEP in expert hands. However, RASP requires longer operative times, access to robotic platforms, and is generally costlier. Studies like that of Autorino et al. have supported its safety profile, but data on oncologic outcomes in incidental PCa remain limited.^24^

Minimally Invasive Surgical Therapies (MIST), including Rezūm and UroLift, provide office‐based alternatives with reduced invasiveness but are unsuitable for large glands and cannot retrieve tissue for cancer evaluation. Consequently, MIST techniques are not advisable in patients with known or suspected PCa.^25^

In summary, while several surgical modalities are available for BPH treatment (Table 3), HoLEP remains uniquely positioned for patients with known or suspected prostate cancer due to its excellent haemostatic profile, ability to manage large prostates and the critical advantage of obtaining diagnostic tissue.

**TABLE 3:** Comparison table of HoLEP versus other common BPH procedures, focusing on bleeding risk and prostate cancer considerations.

Procedure	Bleeding risk	Suitable for prostate cancer	Prostate size suitability	Tissue sample for pathology	Additional notes
**HoLEP**	Low to moderate *(↑ if cancer present or large gland)*	Yes – can relieve obstruction even in cancer	Small to very large (>100 cc)	Yes – complete enucleated tissue available	Excellent tissue removal; pathology available; operator‐dependent
**TURP**	Moderate to high	Sometimes – limited in cancer	Small to medium (<80 cc)	Yes – resected chips available	Higher bleeding risk; shorter learning curve; risk of TUR syndrome
**GreenLight Laser (PVP)**	Low	Not ideal – vaporizes tissue, no pathology	Small to medium	No – tissue is vaporized	Minimal bleeding; cannot retrieve tissue for histology
**Aquablation**	Moderate *(↑ in large glands)*	Not routinely used in cancer	Medium to large	Yes – limited samples	Automated; good for large glands; pathology possible; bleeding manageable
**Open/Robot‐assisted Simple Prostatectomy**	High	Rarely – usually for benign only	Very large (>150 cc)	Yes – entire adenoma	

Limitations of this study included its retrospective design and potential selection bias, although the use of consecutive patients and matched controls aimed to minimize confounding. The study also lacked long‐term follow‐up data on oncological outcomes and functional results, which should be addressed in future prospective studies.

## 5 CONCLUSIONS

This multicentre experience demonstrates that HoLEP in patients with prostate cancer is associated with a higher incidence of bleeding complications compared to non‐cancer patients. The bleeding risk is particularly elevated in those with a preoperative diagnosis of PCa and in patients receiving anticoagulant therapy. Awareness of these risks is important for preoperative planning and patient counselling. Despite this, HoLEP remains an effective and generally safe treatment for lower urinary tract symptoms in patients with PCa, offering the dual benefit of symptom relief and tissue diagnosis.

## AUTHOR CONTRIBUTIONS

**Angelo Porreca:** supervision, validation. **Davide De Marchi:** project administration, validation. **Filippo Marino:** investigation, methodology, software, writing – review and editing. **Marco Giampaoli:** investigation. **Francesca Simonetti:** data curation, formal analysis, software. **Antonio Amodeo:** supervision, visualization. **Paolo Corsi:** investigation, methodology. **Alessandro Crestani:** resources, visualization. **Gian Maria Busetto:** supervision, validation. **Daniele D'Agostino:** resources. **Daniele Romagnoli:** resources. **Luca Di Gianfrancesco:** conceptualization, data curation, formal analysis, investigation, methodology, writing – original draft.

## CONFLICT OF INTEREST STATEMENT

The authors declared that the research was conducted in the absence of any commercial or financial relationships that could be construed as a potential conflict of interest.
